# Visual Sensory Cues and Customer Experience Management in Coffee Shops: An Experimental Study of Gender as a Moderator

**DOI:** 10.12688/f1000research.183621.1

**Published:** 2026-06-17

**Authors:** Nosi Nosi, Tantri Yanuar Rahmat Syah, Triyono Arief Wahyudi, Adilla Anggraeni

**Affiliations:** 1 Universitas Esa Unggul, West Jakarta, Jakarta, Indonesia; 2 Bina Nusantara University, West Jakarta, Jakarta, Indonesia

**Keywords:** visual sensory cues; sensory brand experience; customer experience management; gender; coffee shop; sensory marketing; experimental design

## Abstract

**Background:**

Coffee shops increasingly compete through managed customer experiences rather than product quality alone. Visual sensory cues, including interior color, lighting, decoration, spatial aesthetics, product presentation, and menu design, shape how consumers interpret a service environment. This study examined whether gender moderates the effect of visual sensory cues on sensory brand experience in coffee shop consumption.

**Methods:**

A 2 x 2 between-subjects experiment was conducted by manipulating visual sensory cues into strong and weak conditions and comparing female and male participants. A total of 120 coffee shop consumers participated, with 30 participants assigned to each experimental cell. Manipulation-check items were used to confirm whether participants perceived the intended difference between visual cue conditions. The main hypothesis was tested using a univariate two-way ANOVA/general linear model.

**Results:**

Manipulation checks confirmed that participants distinguished strong from weak visual cue conditions. The interaction between gender and visual sensory cues significantly influenced sensory brand experience (b = 2.191, SE = 0.128, t = 17.091, p < 0.001), supporting the moderation hypothesis. However, female consumers did not report higher sensory brand experience under weak visual cues, and strong visual cues did not significantly increase male consumers’ sensory brand experience.

**Conclusions:**

The findings suggest that sensory design effects are contingent rather than universal. Stronger visual cues do not automatically create stronger sensory brand experience; instead, customer experience management in coffee shops depends on the fit between environmental cues, segment characteristics, and consumer perceptual processing.

## Introduction

Coffee shops have moved beyond a product-centered business model and increasingly compete as experience-centered service spaces ( Said & Tiong, 2023). Consumers do not evaluate coffee shops only through coffee taste, price, location, or service speed; they also respond to atmosphere, visual setting, emotional comfort, and the symbolic meaning created by the brand environment ( Jolliffe, 2010). This makes the coffee shop an appropriate context for examining how customer experience is managed through environmental design.

In experiential and sensory marketing, consumers respond to brands through sensory, affective, cognitive, and behavioral experiences that emerge during their interaction with brand-related stimuli ( Schmitt, 1999; Brakus et al., 2009; Krishna, 2012). Among these stimuli, visual sensory cues are particularly important because vision is often the first sensory channel through which consumers evaluate a service environment ( Turley & Milliman, 2000). Color, lighting, decoration, product display, packaging aesthetics, menu design, and spatial arrangement can shape initial impressions of quality, comfort, authenticity, value, and brand personality ( Yu et al., 2020; Bajaj & Bond, 2017; Wilms & Oberfeld, 2018).

For coffee shop managers, visual design is not merely a matter of decoration. It is a managerial instrument used to communicate atmosphere, positioning, and service quality before direct product evaluation occurs ( Nosi et al., 2026). A visually coherent coffee shop can guide attention, support comfort, stimulate emotion, and create an experiential frame through which coffee aroma, taste, and service interaction are interpreted. Conversely, weak or inconsistent visual cues may reduce processing fluency and weaken the experiential value of the visit.

However, the effect of visual sensory cues may not be identical across consumers ( Pine & Gilmore, 1998). Gender-based information processing literature suggests that women and men may differ in the way they attend to, elaborate, and interpret visual information ( Meyers-Levy, 1989; Meyers-Levy & Sternthal, 1991). Women are often described as more elaborative processors who attend to multiple details, while men are often described as more selective processors who focus on salient and task-relevant cues. These differences imply that the same coffee shop visual environment may generate different sensory brand experiences depending on the consumer group ( Zha et al., 2024).

Despite the importance of sensory design in customer experience management, prior studies have often relied on cross-sectional survey designs. Such studies are valuable for explaining associations, but they are less suited to testing the causal effect of manipulated sensory stimuli. Experimental evidence is therefore needed to determine whether different visual cue conditions produce different levels of sensory brand experience and whether this effect depends on gender ( Nosi et al., 2025).

This study addresses this gap by using a 2 x 2 between-subjects experimental design in which visual sensory cues are manipulated into strong and weak conditions and compared across female and male consumers. The study contributes to sensory marketing, consumer behavior, and service management research by explaining how visual environment management, gender-based segmentation, and customer experience formation interact in coffee shop consumption.

The study makes three contributions. First, it extends sensory marketing theory by showing that visual cue effects are contingent rather than universal. Second, it strengthens the role of gender as a perceptual moderator in customer experience management. Third, it offers practical guidance for coffee shop managers who need to design visual environments that fit the perceptual needs of their target segments rather than assuming that stronger visual cues always produce stronger experiences.

### Theoretical framework and hypotheses

**Visual sensory cues in coffee shop experience management**

Visual sensory cues refer to brand-related stimuli that consumers perceive visually, including color, lighting, interior design, spatial layout, decoration, product display, packaging, typography, menu design, and other aesthetic elements. In coffee shops, these cues are highly visible because the customer usually forms an impression of the brand before ordering, consuming, or evaluating the product directly ( Wang et al., 2024).

The atmospheric perspective in services explains that physical surroundings influence consumers by shaping perception, emotion, and behavioral intention ( Wedel & Pieters, 2008). Visual cues are therefore part of the service evidence that helps consumers interpret whether a coffee shop feels warm, premium, professional, comfortable, creative, or authentic. In this sense, visual cues become a managerial resource for creating customer experience, not merely a design accessory.

A stronger visual cue condition can be understood as an environment with clearer, richer, and more coherent aesthetic signals. A weaker visual cue condition can be understood as an environment with less prominent, less differentiated, or less emotionally engaging visual signals. The managerial question is not only whether strong visual cues are better, but whether the cue intensity matches how consumers process and evaluate the coffee shop environment.

**Sensory brand experience**

Brand experience refers to consumers’ subjective internal responses generated by brand-related stimuli ( Brakus et al., 2009). Sensory brand experience is the sensory dimension of this response, reflecting how a brand is experienced through sight, smell, taste, sound, and touch ( Mehrabian & Russell, 1974). In a coffee shop, sensory brand experience emerges from the interaction between the physical environment, the product, the service process, and the consumer’s personal interpretation.

Sensory brand experience should not be treated as a passive reaction to external stimuli. The same visual cue may be interpreted differently depending on consumer expectations, prior experience, attention, and perceptual style ( Li et al., 2025). This makes sensory brand experience especially relevant for management research because it connects environmental design decisions with psychological and behavioral outcomes.

**Gender as a moderator**

Gender is theoretically relevant because it may shape how consumers process visual information ( Gao & Shen, 2024). The selectivity hypothesis proposes that women tend to engage in more comprehensive information processing, whereas men tend to process information more selectively by relying on salient cues ( Meyers-Levy, 1989; Meyers-Levy & Sternthal, 1991). In visual service environments, these differences may influence how consumers notice details, evaluate coherence, and transform visual information into sensory experience ( Park et al., 2013).

This study therefore treats gender not merely as a demographic descriptor, but as a cognitive and perceptual mechanism that may alter the effectiveness of visual sensory cues. In the coffee shop context, gender may influence whether a visual environment is experienced as rich, comfortable, excessive, insufficient, or functionally clear.

**Hypothesis development**

Sensory marketing theory suggests that visual cues can enhance consumer perception and experience when they provide meaningful, coherent, and emotionally engaging brand stimuli ( Hultén, 2011; Krishna, 2012; Spence, 2020). However, gender-based information processing suggests that the strength of this effect may differ between female and male consumers. Thus, the relationship between visual sensory cues and sensory brand experience is expected to depend on gender.

H1.
*Gender moderates the relationship between visual sensory cues and sensory brand experience.*

Women are often assumed to be more attentive to visual and aesthetic details. Therefore, under weak visual conditions, female consumers were initially expected to show stronger sensory brand experience because they may still be able to identify and elaborate the available visual cues. This leads to the following sub-hypothesis.

H1a.
*Under weak visual sensory cue conditions, female consumers have higher sensory brand experience than male consumers.*

Men are often described as more selective processors who respond to salient and clearly differentiated cues. Stronger visual cues were therefore expected to become more noticeable and easier to process for male consumers than weaker cues. This leads to the following sub-hypothesis.

H1b.
*Among male consumers, strong visual sensory cues produce higher sensory brand experience than weak visual sensory cues.*

## Methods

### Study design

This study used a quantitative experimental approach because the objective was to examine the causal effect of manipulated visual sensory cues on sensory brand experience while considering the moderating role of gender ( Aguinis & Bradley, 2014; Field, 2017). The research employed a 2 x 2 between-subjects factorial design. The first factor was gender, consisting of female and male participants. The second factor was visual sensory cue condition, consisting of strong and weak visual stimuli. The balanced experimental structure is shown in Table 1.

**Table 1.** Experimental design.

Visual sensory cue condition	Female participants	Male participants	Total
Strong visual cues	30	30	60
Weak visual cues	30	30	60
Total	60	60	120

*Note*: Each participant was assigned to one experimental condition only.

A between-subjects design was used because each participant was exposed to only one experimental condition. This design reduced carry-over effects and helped maintain internal validity by preventing respondents from comparing multiple treatment conditions.

### Study setting and participants

The participants were coffee shop consumers from a university student population. The experiment was conducted at Universitas Banten Jaya. A total of 120 participants took part in the experiment and were divided equally into four experimental cells, with 30 participants in each cell. Participants were screened to ensure that they had experience consuming coffee in coffee shops, with a minimum consumption frequency of twice per week.

The sample size was considered appropriate for a balanced 2 x 2 experimental design because each cell contained an equal number of participants, allowing comparison of treatment conditions and interaction effects.

### Stimuli, materials, and procedure

The independent variable was visual sensory cue condition. The strong visual condition represented a coffee shop environment with more salient and coherent visual cues, including stronger color arrangement, more visible lighting effects, richer decorative elements, clearer menu presentation, and more attractive beverage or product presentation. The weak visual condition represented a less salient visual environment with fewer visual details, less prominent aesthetic treatment, and lower visual intensity.

The manipulation focused on the visual characteristics that are commonly used in coffee shop branding: interior color, lighting, decoration, spatial aesthetics, product presentation, packaging appearance, and menu design. A manipulation check was conducted to verify whether participants perceived the intended difference between strong and weak visual cue conditions.

The experimental procedure consisted of participant recruitment, screening, assignment to experimental conditions, exposure to the visual stimulus, manipulation check, and sensory brand experience measurement. Before participating in the experiment, all participants received information about the study and provided written informed consent. Participants were first screened according to coffee shop consumption experience. They were then assigned to one of the four experimental cells and exposed to the assigned visual sensory cue condition. After exposure, participants completed the manipulation-check items and the sensory brand experience scale.

### Measures and instruments

Sensory brand experience was measured using Likert-type items adapted from the brand experience literature, particularly the sensory dimension of brand experience. The item scores were processed into a composite score representing sensory brand experience for hypothesis testing. Visual sensory cue perception was measured through manipulation-check items labeled VSC1, VSC2, and VSC3.

Gender was coded as a categorical moderator. Visual cue condition was also coded as a categorical factor. The interaction between gender and visual cue condition was included to test whether gender moderated the effect of visual sensory cues on sensory brand experience.

The questionnaire, stimulus descriptions, and variable coding are provided as extended data so that readers can evaluate item wording, scale anchors, and replication procedures.

### Pilot testing and reliability

The visual manipulation and questionnaire items were reviewed for face validity before administration. Manipulation-check items were used to evaluate whether the stimuli were perceived as intended, and the sensory brand experience items were processed into a composite score for hypothesis testing.

### Bias control and quality checks

The study used a balanced between-subjects structure, with one exposure per participant, to reduce carry-over and comparison effects. Manipulation-check items were used to confirm whether the experimental treatment was perceived as intended. No personally identifiable information was analyzed, and responses were reported only in aggregate form.

Levene statistics were examined to assess the homogeneity of variance assumption for the manipulation-check items. The main outcome model was checked using the same data-quality logic, including inspection of missing data and any exclusion rules before final analysis.

### Data analysis

The manipulation check was analyzed using ANOVA to determine whether participants perceived significant differences between strong and weak visual cue conditions. Levene statistics were also examined to assess the homogeneity of variance assumption.

The hypothesis was tested using a general linear model/univariate two-way ANOVA approach. The model examined the effect of gender, visual sensory cue condition, and the interaction between gender and visual sensory cue condition on sensory brand experience. The parameter estimate model was specified as follows:

Y=β0+β1X1+β2X2+β3X1X2+e

where Y represents sensory brand experience, X1 represents gender, X2 represents visual sensory cue condition, and X1X2 represents the interaction term.

### Preregistration statement

This study and its analysis plan were not preregistered. The design, procedure, measures, and analysis strategy are reported transparently to support reproducibility.

## Results

### Participant profile and descriptive statistics

A total of 120 coffee shop consumers participated in the experiment. The design was balanced across gender and visual cue conditions, with 30 participants in each experimental cell ( Table 1).

### Manipulation checks

The manipulation check confirmed that the visual stimulus was perceived as intended. The ANOVA significance values for the three manipulation-check items were below 0.001, indicating that participants clearly distinguished strong and weak visual sensory cue conditions ( Hauser et al., 2018). The Levene statistics also suggested that the homogeneity assumption was acceptable for the manipulation-check items ( Table 2).

**Table 2.** Manipulation check for visual sensory cue conditions.

Manipulation item	Levene statistic	ANOVA significance
VSC1	0.551	< 0.001
VSC2	0.164	< 0.001
VSC3	0.282	< 0.001

*Note*: VSC = visual sensory cue. Values are based on the experimental manipulation check.

### Hypothesis testing/main analysis

The hypothesis testing showed that the interaction between gender and visual sensory cues significantly influenced sensory brand experience. The interaction parameter [Gender = 1] x [Visual = 1] was positive and significant (b = 2.191, SE = 0.128, t = 17.091, p < 0.001). This result supports H1 and indicates that gender moderates the relationship between visual sensory cues and sensory brand experience ( Table 3).

**Table 3.** Parameter estimates for the gender x visual sensory cue model.

Parameter	b	SE	t	p-value	Interpretation
Intercept	0.530	0.064	8.265	< 0.001	Baseline estimate
Gender = 1	−2.147	0.091	−23.694	< 0.001	Gender effect in the specified coding
Visual = 1	−0.007	0.091	−0.081	0.936	Visual cue effect in the specified coding
Gender = 1 x Visual = 1	2.191	0.128	17.091	< 0.001	Moderation effect

*Note*: SE = standard error. The table reports the parameter estimates available from the experimental analysis.

The main effect of visual sensory cues in the reported parameterization was not significant (b = −0.007, SE = 0.091, t = −0.081, p = 0.936). This means that stronger visual cues did not automatically produce higher sensory brand experience across the tested comparison. The result challenges the simple assumption that increasing visual intensity will always improve customer experience.

H1a was not supported. Under weak visual cue conditions, female consumers did not report higher sensory brand experience than male consumers. The direction of the finding indicated the opposite pattern: male respondents reported a more positive sensory brand experience under weak visual stimuli.

H1b was also not supported. Among male respondents, the sensory brand experience score under strong visual cues was 0.522, whereas under weak visual cues it was 0.530. The difference was very small and statistically insignificant (b = −0.007, p = 0.936). Thus, stronger visual cues did not significantly improve sensory brand experience among male consumers. The overall hypothesis decisions are summarized in Table 4.

**Table 4.** Summary of hypothesis testing.

Hypothesis	Statement	Result
H1	Gender moderates the relationship between visual sensory cues and sensory brand experience.	Supported
H1a	Under weak visual cue conditions, female consumers have higher sensory brand experience than male consumers.	Not supported
H1b	Among male consumers, strong visual cues produce higher sensory brand experience than weak visual cues.	Not supported

### Additional/robustness analysis

No additional subgroup, mediation, or robustness analysis was conducted in this study. Future research may include sensitivity checks or alternative coding of the categorical variables to further examine the stability of the findings.

## Discussion

### Summary of key findings

The findings show that gender is an important moderator in the relationship between visual sensory cues and sensory brand experience. This means that the effect of coffee shop visual design cannot be understood as a uniform stimulus-response mechanism. Consumers interpret visual cues through perceptual filters, expectations, and information-processing tendencies. Therefore, visual design functions as a managerial instrument whose effectiveness depends on the target consumer segment.

The supported moderation effect strengthens the argument that sensory marketing should move beyond universal design assumptions. A visual environment that is attractive to one segment may not create the same experience for another segment. In coffee shop management, visual cues such as lighting, color, layout, and product display should be designed not only to look aesthetically strong, but also to fit how consumers process the environment.

The unsupported H1a is theoretically meaningful. Although women are often assumed to be more sensitive to visual and aesthetic details, higher sensitivity does not necessarily produce higher sensory brand experience under weak visual conditions. Instead, higher sensitivity may make insufficient or incoherent visual cues easier to detect. This finding suggests a sensory sensitivity paradox: consumers who are more attentive to sensory details may evaluate the experience more critically when the stimulus lacks richness or coherence ( Berlyne, 1960, 1971; Bitner, 1992).

The unsupported H1b also provides insight into male consumer responses. Stronger visual cues did not significantly improve sensory brand experience among male respondents. This suggests that men in this experimental context may not respond simply to visual intensity. Their experience may depend more on visual relevance, functionality, seating comfort, spatial clarity, and ease of use than on richer aesthetic details alone ( Cohen et al., 2003).

Taken together, the findings suggest that sensory brand experience is nonlinear and contingent. Stronger visual cues are not automatically better. Visual design may produce diminishing returns if it exceeds consumers’ preferred level of stimulation or if it adds complexity without increasing perceived relevance. Conversely, weak visual cues may not be neutral; they may be interpreted as insufficient, especially by consumers who are more sensitive to aesthetic details.

### Theoretical implications

First, this study extends sensory marketing theory by showing that visual sensory cue effects are contingent rather than universal. The significant interaction indicates that customer experience emerges from the fit between environmental stimuli and consumer characteristics.

Second, the study strengthens gender as a moderator in sensory brand experience research. Gender should not be treated only as a control variable. It can operate as a perceptual and cognitive mechanism that shapes how visual cues are noticed, interpreted, and evaluated ( Bairrada et al., 2019).

Third, the study challenges the linear intensity assumption in sensory marketing. The finding that strong visual cues did not significantly increase male consumers’ sensory brand experience suggests that customer experience may follow an optimal-stimulation logic rather than a simple stronger-is-better logic.

Fourth, this study contributes to coffee shop branding research by linking visual environment management, gender-based segmentation, and sensory brand experience in an experimental design. This connection translates consumer psychology into service management decision-making.

### Managerial implications

For coffee shop managers, the findings suggest that visual design should not be developed only according to general aesthetic preferences. Managers should first identify the dominant customer segment and the type of experience the brand wants to create. A coffee shop targeting consumers who value aesthetic richness may need warmer colors, softer lighting, meaningful decorative details, and visually attractive product presentation. However, these cues should remain coherent and not become visually excessive ( Yerkes & Dodson, 1908).

For male-oriented or functionally oriented segments, managers should avoid assuming that more visual complexity will automatically increase experience. The visual strategy may be more effective when it emphasizes clarity, spatial order, comfortable seating, easy menu reading, appropriate lighting, and a clean interior layout.

For mixed-gender coffee shop markets, the best strategy is likely a balanced design that combines aesthetic engagement and functional clarity. The environment should be visually attractive enough to create emotional attachment, but also simple enough to support processing fluency, comfort, and service convenience. Managers should therefore conduct periodic customer feedback or small experimental tests before implementing major visual redesigns.

The findings also imply that coffee shop managers should view sensory design as part of customer experience management. Visual cues should be aligned with aroma, taste, service interaction, and brand positioning. A visually attractive shop that lacks product consistency may fail to create loyalty, while a good product in a weak visual environment may not produce a strong brand experience.

### Strengths and limitations

The main strength of this study is its experimental design, which allows a clearer test of visual cue effects than a purely cross-sectional survey. The balanced 2 x 2 structure also supports comparison across gender and visual cue conditions.

This study has several limitations. First, the sample consisted of coffee shop consumers from a university student population, which may limit generalizability to broader consumer groups. Second, the study focused on visual sensory cues only, although coffee shop experience is multisensory. Third, the experiment used strong and weak visual cue conditions; more nuanced design styles were not tested.

### Future research

Future research should test the model across different age groups, cities, and coffee shop segments. Studies may also examine visual cues together with olfactory, taste, auditory, and tactile cues to explain multisensory experience more comprehensively.

Further experiments may test more nuanced visual design levels, such as minimalist, warm, premium, natural, industrial, or digitally enhanced atmospheres. Future research should also include downstream outcomes such as satisfaction, brand love, brand respect, willingness to pay, revisit intention, and brand loyalty to explain how sensory brand experience becomes managerial value over time.

## Conclusions

This study concludes that gender moderates the relationship between visual sensory cues and sensory brand experience in the coffee shop context. The results show that visual sensory cues do not influence all consumers in the same way. Instead, sensory brand experience is shaped by the interaction between environmental design and consumer perceptual processing.

The findings also show that stronger visual cues do not always create stronger sensory brand experience. H1a and H1b were not supported, indicating that female consumers did not report higher experience under weak visual cues and male consumers did not report higher experience under strong visual cues. These findings challenge simple linear assumptions in sensory marketing and suggest that sensory design effectiveness depends on segment fit, stimulus coherence, and optimal stimulation.

For service and consumer experience research, the study demonstrates that customer experience management in coffee shops requires an integrated understanding of design, psychology, gender-based segmentation, and service strategy. The key managerial question is not simply how strong the visual stimulus should be, but whether the visual environment fits the consumers for whom the brand experience is designed.

## Ethics and consent

Ethical approval for this study was obtained from the Research Ethics Committee of Universitas Esa Unggul, Indonesia (approval number: No. 0925–05.061/DPKE-KEP/FINAL-EA/UEU/V/2026; approval date: 19 May 2026). All participants provided written informed consent before taking part in the study. Prior to participation, participants were informed about the purpose of the study, the voluntary nature of their involvement, the confidentiality of their responses, and their right to withdraw from the study at any time without penalty. No personally identifiable information was collected or analyzed, and all responses were reported only in aggregate form.

## Generative AI statement

During the preparation of this manuscript, generative AI assistance was used only to support language editing, clarity improvement, formatting alignment, and journal-scope adjustment. The authors reviewed and edited the output and take full responsibility for the content of the publication.

**Abbreviations**
SBE: Sensory brand experience
VSC: Visual sensory cues

## Data Availability

The underlying data for this study are publicly available in Zenodo: https://doi.org/10.5281/zenodo.20404584 Nosi, N., Syah, T. Y. R., Wahyudi, T. A., & Anggraeni, A. (2026). Visual Sensory Cues and Customer Experience Management in Coffee Shops: An Experimental Study of Gender as a Moderator [Data set]. Zenodo. https://doi.org/10.5281/zenodo.20404584 This project contains the following underlying data: anonymised respondent-level dataset, variable codebook, manipulation-check data, and analysis output used for the ANOVA/general linear model. The data are publicly available under the terms of the selected repository licence. The extended data for this study are available in the same Zenodo record: https://doi.org/10.5281/zenodo.20404584. The extended materials include the questionnaire, visual stimulus descriptions, manipulation materials, participant information sheet, informed consent form, and methodological transparency/reporting checklist used for the study. The extended data are publicly available under the terms of the selected repository licence. Data analysis was conducted using IBM SPSS Statistics 26. No custom software or code was used. No formal reporting guideline is mandated for this experimental consumer research article. To support transparency, the extended data repository includes a concise methodological transparency file covering the study design, participant screening, stimuli, manipulation check, measures, analysis model, and ethics details.
