# Supplementary material for: Visual Sensory Cues and Customer Experience Management in Coffee Shops: An Experimental Study of Gender as a Moderator
Source: F1000Res. 2026 Jun 17;15:955. [Version 1] doi: 10.12688/f1000research.183621.1 (PMC13309771; doi:10.12688/f1000research.183621.1)
Supplement: Peer review history

# Reviewer report: Reviewer response for version 1

**DOI:** 10.5256/f1000research.202679.r495588

**Published:** 2026-06-26

**Authors:** Meilisa Alvita

**Affiliations:** 1 Universitas Pradita, Tangerang, Indonesia

Overall, the research background is appropriate and clearly explains the urgency and relevance of the study. The research method used is also suitable and able to address the research objectives effectively. This article provides a valuable contribution to the development of knowledge, particularly in the related field of study is good.

Is the work clearly and accurately presented and does it cite the current literature?

Yes

If applicable, is the statistical analysis and its interpretation appropriate?

Yes

Are all the source data underlying the results available to ensure full reproducibility?

Yes

Is the study design appropriate and is the work technically sound?

Yes

Are the conclusions drawn adequately supported by the results?

Yes

Are sufficient details of methods and analysis provided to allow replication by others?

Yes

Reviewer Expertise:

marketing research as a lecturer

I confirm that I have read this submission and believe that I have an appropriate level of expertise to confirm that it is of an acceptable scientific standard.

# Reviewer report: Reviewer response for version 1

**DOI:** 10.5256/f1000research.202679.r495584

**Published:** 2026-06-25

**Authors:** Rohaelis Nuraisiah

**Affiliations:** 1 Universitas Banten Jaya, Jl Syech Nawawi Albantani Serang, Serang, Banten, Indonesia

Overall, this article presents a relevant and valuable contribution to the field. The topic is clearly aligned with current academic discussions, and the manuscript demonstrates a coherent structure, a clear research objective, and an appropriate connection between the theoretical background, methodology, findings, and discussion.

 The article provides a meaningful explanation of the research problem and offers useful insights for both academic and practical contexts. The methodology is generally appropriate for addressing the research objectives, and the findings are presented in a clear and understandable manner. The discussion also reflects the authors’ ability to relate the results to the existing literature and to explain the contribution of the study.

 In my view, the article is scientifically sound. The main arguments are logically developed, the research design is acceptable, and the conclusions are supported by the findings. No major issues were identified that would undermine the scientific quality of the article.

 Therefore, I consider the article suitable for publication. The manuscript makes a positive contribution and can be accepted in its current form.

Is the work clearly and accurately presented and does it cite the current literature?

Yes

If applicable, is the statistical analysis and its interpretation appropriate?

Yes

Are all the source data underlying the results available to ensure full reproducibility?

Yes

Is the study design appropriate and is the work technically sound?

Yes

Are the conclusions drawn adequately supported by the results?

Yes

Are sufficient details of methods and analysis provided to allow replication by others?

Yes

Reviewer Expertise:

My areas of research expertise include Marketing Management, Consumer Behavior, Sensory Marketing, Brand Experience, Customer Experience Management, Branding, Brand Love, Brand Satisfaction, and Brand Loyalty. I am also familiar with quantitative research methods, survey-based studies, and consumer response analysis in service and retail contexts.

I confirm that I have read this submission and believe that I have an appropriate level of expertise to confirm that it is of an acceptable scientific standard.

# Response

**Published:** 2026-06-25

**Authors:** Nosi Nosi

**Affiliations:** Management, Universitas Esa Unggul, West Jakarta, Jakarta, Indonesia

**Thank you very much for your review. It truly encourages me to keep contributing and bringing value to others.**
